# Cerebral Hemodynamics as a Diagnostic Bridge Between Mild Cognitive Impairment and Late-Life Depression: A Multimodal Approach Using Transcranial Doppler and MRI

**DOI:** 10.3390/life15081246

**Published:** 2025-08-06

**Authors:** Sergiu-Florin Arnautu, Diana-Aurora Arnautu, Minodora Andor, Cristina Vacarescu, Dragos Cozma, Brenda-Cristina Bernad, Catalin Juratu, Adrian Tutelca, Catalin-Dragos Jianu

**Affiliations:** 1 Centre for Cognitive Research in Neuropsychiatric Pathology (NeuroPsy-Cog), Department of Neuro-Sciences, Victor Babes University of Medicine and Pharmacy, E. Murgu Sq., No. 2, 300041 Timisoara, Romania; arnautu.sergiu@umft.ro (S.-F.A.); jianu.dragos@umft.ro (C.-D.J.); 2 Department of Internal Medicine I, Faculty of Medicine, Victor Babes University of Medicine and Pharmacy, 2nd Eftimie Murgu Square, 340001 Timisoara, Romania; 3 Cardiology Clinics, Timisoara Clinical Municipal Emergency Hospital, Bd. Revolutiei din 1989, No. 12, 300041 Timisoara, Romania; 4 Department of Cardiology, Faculty of Medicine, Victor Babes University of Medicine and Pharmacy, 2nd Eftimie Murgu Sq., 300041 Timisoara, Romaniadragoscozma@gmail.com (D.C.); 5 Institute of Cardiovascular Diseases Timisoara, 13A Gheorghe Adam Street, 300310 Timisoara, Romania; 6 Doctoral School, Victor Babes University of Medicine and Pharmacy, Eftimie Murgu Square 2, 300041 Timisoara, Romania; bernad.brenda@umft.ro (B.-C.B.);; 7 Center for Neuropsychology and Behavioral Medicine, Victor Babes University of Medicine and Pharmacy, 300041 Timisoara, Romania; 8 Department of Radiology, County Clinical Emergency Hospital, 156 L. Rebreanu Ave., 300736 Timisoara, Romania; atutelca@gmail.com; 9 Neurology Clinics II, County Clinical Emergency Hospital, 156 L. Rebreanu Ave., 300736 Timisoara, Romania

**Keywords:** late-life depression, mild cognitive impairment, transcranial Doppler ultrasonography, cerebral hemodynamics, vascular cognitive impairment, white matter lesions, cognitive decline, neurovascular dysfunction

## Abstract

Background: Vascular dysfunction is increasingly recognized as a shared contributor to both cognitive impairment and late-life depression (LLD). However, the combined diagnostic value of cerebral hemodynamics, neuroimaging markers, and neuropsychological outcomes remains underexplored. This study aimed to investigate the associations be-tween transcranial Doppler (TCD) ultrasound parameters, cognitive performance, and depressive symptoms in older adults with mild cognitive impairment (MCI) and LLD. Importantly, we evaluated the integrative value of TCD-derived indices alongside MRI-confirmed white matter lesions (WMLs) and standardized neurocognitive and affective assessments. Methods: In this cross-sectional study, 96 older adults were enrolled including 78 cognitively unimpaired individuals and 18 with MCI. All participants underwent structured clinical, neuropsychological, and imaging evaluations including the Mini-Mental State Examination (MMSE), Montreal Cognitive Assessment (MoCA), Geriatric Depression Scale (GDS-15), MRI-based Fazekas scoring of WMLs, and TCD ultrasonography of the middle cerebral artery. Hemodynamic variables included mean blood flow velocity (MBFV), end-diastolic velocity (EDV), pulsatility index (PI), and resistive index (RI). Logistic regression and receiver operating characteristic (ROC) analyses were used to identify independent predictors of MCI. Results: Participants with MCI showed significantly lower MBFV and EDV, and higher PI and RI (*p* < 0.05 for all) compared with cognitively unimpaired participants. In multivariate analysis, lower MBFV (OR = 0.64, *p* = 0.02) and EDV (OR = 0.70, *p* = 0.03), and higher PI (OR = 3.2, *p* < 0.01) and RI (OR = 1.9, *p* < 0.01) remained independently associated with MCI. ROC analysis revealed excellent discriminative performance for RI (AUC = 0.919) and MBFV (AUC = 0.879). Furthermore, PI correlated positively with depressive symptom severity, while RI was inversely related to the GDS-15 scores. Conclusions: Our findings underscore the diagnostic utility of TCD-derived hemodynamic parameters—particularly RI and MBFV—in identifying early vascular contributions to cognitive and affective dysfunction in older adults. The integration of TCD with MRI-confirmed WML assessment and standardized cognitive/mood measures represents a novel and clinically practical multi-modal approach for neurovascular profiling in aging populations.

## 1. Introduction

Late-life depression (LLD) is increasingly recognized as a significant risk factor for the development of mild cognitive impairment (MCI) and dementia [1,2]. In addition to its psychiatric manifestations, LLD has been associated with neurodegenerative changes and cerebrovascular pathology. This has led to the concept of “vascular depression”, where vascular dysfunction is thought to contribute to both affective and cognitive symptoms [3]. Structural neuroimaging studies consistently report an increased burden of white matter lesions (WMLs) and subcortical ischemic changes in individuals with LLD, highlighting the role of small vessel disease in the pathophysiology of LLD and related cognitive impairments [4,5].

Vascular cognitive impairment (VCI) encompasses a spectrum of cognitive disorders attributed to cerebrovascular pathology. It is broadly categorized based on severity and underlying vascular mechanisms. Mild VCI is defined by measurable cognitive deficits—commonly in executive function, processing speed, and attention—without significant interference in activities of daily living, whereas vascular dementia (VaD) is characterized by more profound cognitive decline and functional impairment [6,7]. Mild VCI is often viewed as a prodromal stage of VaD, although the trajectory of progression remains variable [8]. Neuroimaging features such as white matter hyperintensities, lacunes, and cerebral microbleeds are commonly observed across the VCI spectrum and tend to be more prominent in VaD [7]. Accurate differentiation between mild VCI and VaD is crucial for prognosis and therapeutic decision-making, and current diagnostic frameworks emphasize a combination of cognitive testing, functional assessment, and neuroimaging findings [9].

VCI may also be classified by etiology into subtypes such as post-stroke dementia, subcortical ischemic vascular disease, multi-infarct dementia, strategic infarct dementia, and mixed dementia, the latter referring to coexisting vascular and neurodegenerative pathology [7]. Small vessel disease, marked by lacunar infarcts and white matter hyperintensities, plays a central role in the milder forms of VCI [8]. Recent guidelines underscore the need for integrated clinical, neuropsychological, and neuroimaging assessments to delineate these subtypes accurately [9].

Transcranial Doppler ultrasound (TCD) is a non-invasive, bedside imaging modality that provides real-time assessment of cerebral hemodynamics by measuring blood flow velocity and pulsatility indices in major intracranial arteries. These parameters offer insight into cerebrovascular resistance and small vessel function—factors implicated in the development of VCI [6,10]. Additionally, TCD allows for the evaluation of vasomotor reactivity and the detection of microembolic signals, both of which have been linked to cognitive decline and an elevated risk of dementia progression [11,12]. Despite limitations such as operator dependency and variability in acoustic windows, TCD remains a valuable tool for assessing cerebrovascular function, particularly in early or subclinical stages.

The primary objective of this study was to evaluate cerebral hemodynamic parameters using TCD in patients with LLD and to examine their associations with cognitive function, specifically the presence of MCI. A secondary aim was to investigate the relationship between TCD-derived indices and the burden of subcortical ischemic vascular disease, as measured by WML severity on structural neuroimaging. By integrating clinical, cognitive, neuroimaging, and hemodynamic assessments, we aimed to explore the potential of TCD as a non-invasive modality for detecting vascular contributions to cognitive impairment in patients with LLD.

Although previous studies have independently explored cerebral hemodynamics in either cognitive impairment or LLD, few have examined both conditions concurrently within the context of MRI-confirmed small vessel disease. Moreover, integrated analyses combining TCD parameters, lesion severity, and standardized cognitive and mood assessments remain rare. The present study addresses this gap by assessing whether TCD-derived vascular indices reflect the burden of both cognitive and affective dysfunction in individuals with LLD and comorbid MCI, stratified by WML severity. We hypothesized that TCD parameters indicative of impaired cerebral perfusion would be associated with greater cognitive and affective dysfunction and a higher burden of white matter lesions in older adults.

## 2. Materials and Methods

### 2.1. Study Design and Participants

This cross-sectional study enrolled elderly patients (aged ≥ 65 years) who presented with depressive symptoms and underwent brain MRI at three major healthcare centers in Timisoara, Romania: the Neurology Ambulatory and Cardiology Departments of the Timisoara Municipal and County Clinical Emergency Hospitals and the Timisoara Institute of Cardiovascular Diseases. Recruitment took place between January 2024 and January 2025.

Sample size was based on feasibility and recruitment capacity during the study period. Given the exploratory nature of the study, our cohort size aligns with comparable neurovascular studies in this field.

Eligible participants exhibited depressive symptoms and MRI-confirmed subcortical ischemic vascular disease, characterized predominantly by white matter lesions (WMLs), and were diagnosed with either normal cognition or mild cognitive impairment (MCI). Inclusion criteria were based on medical history, neurological examination, neuropsychological assessment, and neuroradiological findings.

Exclusion criteria included: a Mini-Mental State Examination (MMSE) score of ≤20 [13]; a history of stroke, transient ischemic attack (TIA), or other neurological disorders such as Parkinson’s disease, epilepsy, or traumatic brain injury; major psychiatric conditions including schizophrenia, obsessive-compulsive disorder, or major depressive disorder; acute or chronic decompensated medical illnesses; endocrine disorders known to affect cognitive function; alcohol or substance abuse; ultrasound evidence of ≥50% stenosis in extracranial or intracranial arteries; bilateral absence of suitable transtemporal acoustic windows for transcranial Doppler (TCD) examination; and any contraindications to undergoing MRI.

All clinical, neuropsychological, and imaging evaluators were blinded to each other’s assessments to minimize potential bias.

### 2.2. Clinical and Demographic Assessment

Demographic and clinical data were collected including age, sex, years of education, cardiovascular and cerebrovascular risk factors (hypertension, diabetes mellitus, hyperlipidemia, smoking, atrial fibrillation, and ischemic heart disease), and personal or family history of depression. Standardized treatment for vascular risk factors was administered to all participants including antiplatelet or anticoagulant agents (aspirin, clopidogrel, or oral anticoagulants); antihypertensive therapy such as angiotensin-converting enzyme (ACE) inhibitors, angiotensin II receptor blockers (ARBs), diuretics, or calcium channel blockers as indicated; lipid-lowering therapy with statins; and antidiabetic agents, including oral hypoglycemics or insulin, based on individual glycemic control needs.

### 2.3. Neuropsychological Assessment

All participants underwent standardized neuropsychological testing conducted by trained neuropsychologists. Global cognitive status was assessed using the Montreal Cognitive Assessment (MoCA) and the Mini-Mental State Examination, 2nd Edition (MMSE-2) [13,14]. The MoCA evaluates domains such as attention, executive function, memory, language, visuoconstruction, abstraction, calculation, and orientation, with a total score of 30; scores between 19 and 25 were considered indicative of mild cognitive impairment (MCI) [15]. The MMSE-2 similarly provides a 30-point measure of cognitive performance across multiple domains. Based on established cutoffs [16], scores were categorized as follows: normal cognition (25–30), mild cognitive impairment (21–24), moderate impairment (10–20), and severe impairment (<10). Diagnosis of MCI was further supported by the VASCOG criteria [7], which emphasize objective deficits in executive function, processing speed, and complex attention, in the context of MRI-confirmed white matter lesions (WMLs) and preserved daily functioning [17,18].

### 2.4. Assessment of Depressive Symptoms

Depressive symptoms were assessed using the 15-item Geriatric Depression Scale (GDS-15) [19], a validated screening tool for depressive symptoms in older adults. The GDS-15 consists of 15 yes/no questions focused on affective symptoms. A score ≥5 was considered indicative of clinically significant depression [19,20].

### 2.5. White Matter Lesion (WML) Assessment

All participants underwent MRI on a 1.5 Tesla General Electric system using standardized sequences including T1-weighted, T2-weighted, proton density-weighted, and fluid-attenuated inversion recovery (FLAIR) images. Scans were acquired with a slice thickness of 5 mm and an interslice gap of 0.5 mm.

The severity of WMLs was rated using the Fazekas scale [17], a widely used visual grading tool for small vessel disease. Fazekas scores were assigned separately for periventricular and deep white matter regions. Periventricular white matter lesions (WMLs) were graded as follows: 0 = absent; 1 = caps or pencil-thin lining; 2 = smooth halo; and 3 = irregular periventricular signal extending into the deep white matter. Deep WMLs were scored as: 0 = absent; 1 = punctate foci; 2 = beginning confluence of foci; and 3 = large confluent areas.

Higher Fazekas scores indicate a more severe burden of small vessel ischemic disease. All MRI scans were independently reviewed by two board-certified neuroradiologists. The inter-rater agreement for Fazekas scores between the two neuroradiologists was excellent (Cohen’s kappa = 0.87). Discrepancies were resolved by consensus.

**Figure 1:**
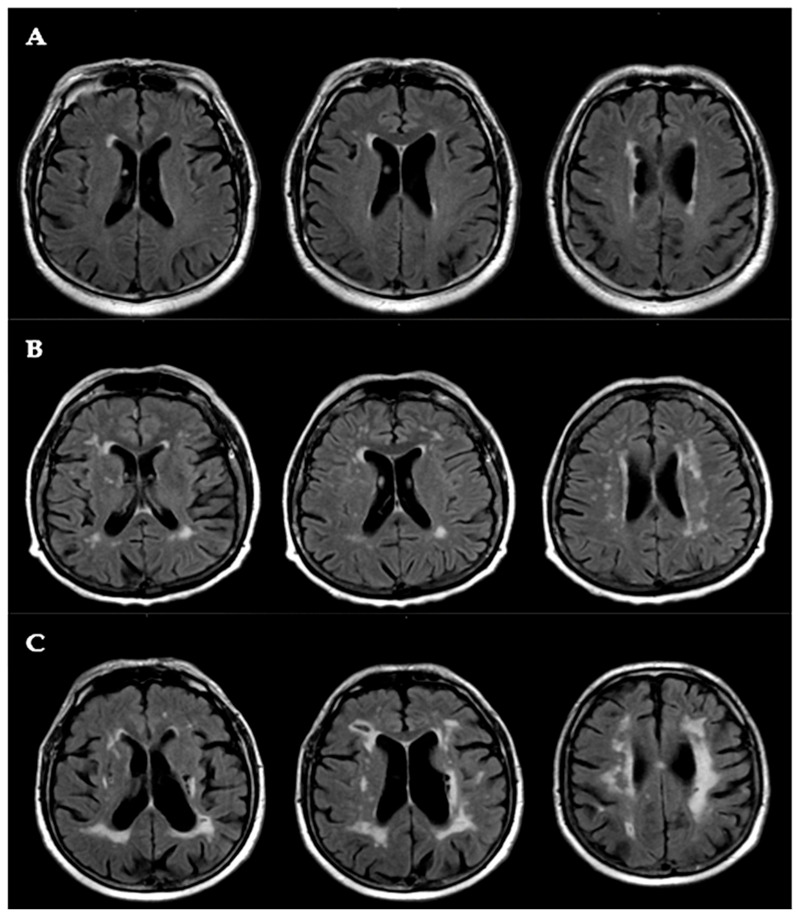
Representative axial fluid-attenuated inversion recovery (FLAIR) MRI images showing varying degrees of white matter lesion (WML) severity, graded according to the Fazekas scale. (**A**) Mild WMLs: punctate hyperintensities; (**B**) moderate WMLs: beginning confluence of lesions; (**C**) severe WMLs: large confluent areas extending into subcortical regions.

Representative axial FLAIR images illustrating WML severity according to the Fazekas classification are shown in Figure 1. Figure 1 displays representative axial fluid-attenuated inversion recovery (FLAIR) MRI images demonstrating the typical distribution and severity of white matter lesions (WMLs) used for lesion grading according to the Fazekas scale.

### 2.6. Transcranial Doppler (TCD) Protocol

**Figure 2:**
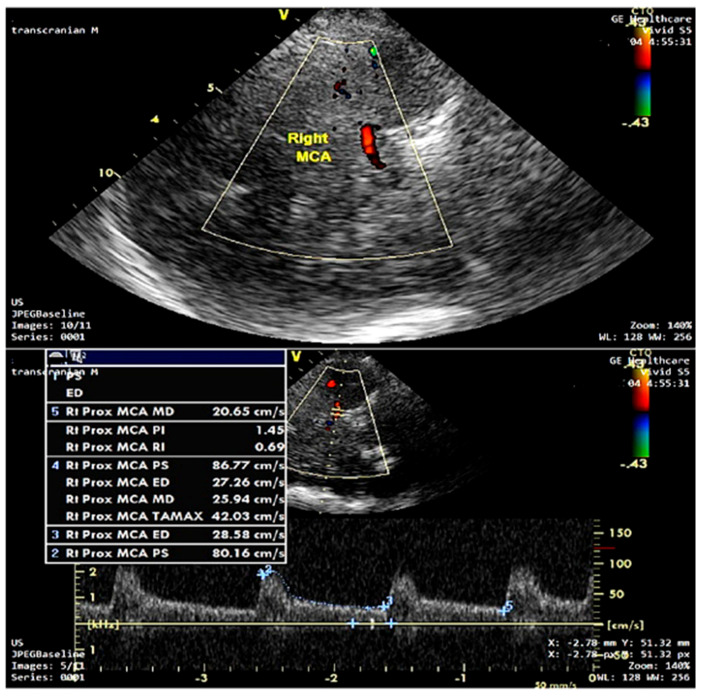
Transcranial Doppler ultrasound recording of the middle right cerebral artery (MCA). The spectrum shows peak systolic velocity (PSV), end-diastolic velocity (EDV), and mean blood flow velocity (MBFV) used for the calculation of the pulsatility index (PI) and resistivity index (RI).

Transcranial Doppler (TCD) sonography was conducted using a General Electric Vivid S5 ultrasound system. All TCD assessments were performed by a single certified neurosonographer experienced in cerebrovascular ultrasound to minimize inter-operator variability. Bilateral recordings of the proximal (M1) segment of the middle cerebral artery (MCA) were obtained via the transtemporal bone window using a 2-MHz pulsed-wave Doppler probe under resting conditions, typically at a depth of 50–60 mm to ensure optimal signal quality. The following hemodynamic parameters were measured: peak systolic velocity (PSV), end-diastolic velocity (EDV), and mean blood flow velocity (MBFV). The pulsatility index (PI) was calculated using the Gosling and King formula: PI = (PSV − EDV)/MBFV [21], and the resistivity index (RI) was calculated using Pourcelot’s method: RI = (PSV − EDV)/PSV [22]. Measurements were obtained after a stable 30 s recording period and averaged over at least 10 consecutive cardiac cycles [23,24]. For each MCA, the mean of two bilateral measurements was recorded; if only one side was accessible, that value was used. In patients with atrial fibrillation, the cardiac cycle showing the highest PSV—indicating the most effective ventricular contraction—was selected for analysis, as recommended [23,25]. Heart rate and mean arterial pressure were recorded simultaneously during TCD assessment. All data were digitally stored for offline analysis. Figure 2 illustrates a representative TCD spectral waveform of the MCA, demonstrating the measurement of PSV, EDV, MBFV, PI, and RI.

### 2.7. Ethics

This study was conducted in accordance with the Declaration of Helsinki and approved by the Ethics Committee of the Municipal Clinical Emergency Hospital, Timisoara. All participants provided written informed consent prior to inclusion in the study.

### 2.8. Statistical Analysis

All statistical analyses were performed using MedCalc Statistical Software version 23.2.6 (MedCalc Software Ltd., Ostend, Belgium; https://www.medcalc.org; 2025, accessed on 1 February 2025) [26]. The sample was stratified into two groups based on cognitive status, and group comparisons were conducted accordingly. The Shapiro–Wilk test was used to assess the normality of continuous variables. Data with a normal distribution are presented as the mean ± standard deviation (SD), while non-normally distributed data are expressed as the median with interquartile range (IQR). Categorical variables are reported as frequencies and percentages. Group differences were assessed using the Student’s *t*-test or Mann–Whitney U test for continuous variables, and the chi-square test or Fisher’s exact test for categorical variables, as appropriate. Logistic regression and receiver operating characteristic (ROC) curve analyses were employed to identify predictors of mild cognitive impairment. Variables with a *p*-value < 0.10 in the univariate analysis were included in the multivariate logistic regression model. There were no dropouts or incomplete datasets; all participants completed the full neuro-psychological, imaging, and TCD protocols.

## 3. Results

### 3.1. Clinical and Demographic Characteristics

A total of 96 participants were included in the analysis, with 78 classified as cognitively unimpaired (Group I) and 18 diagnosed with mild cognitive impairment (Group II). The overall mean age was 71.8 ± 5.5 years. In Table 1, a statistically significant difference in age was observed between groups, with participants in the mild cognitive impairment (MCI) group being older on average than those without cognitive impairment (72.5 ± 5.2 years vs. 68.8 ± 5.8 vs., *p* = 0.01). No significant differences were found between groups in terms of sex distribution, educational level, smoking status, or cardiovascular risk factors including hypertension, hypercholesterolemia, diabetes, coronary artery disease, and atrial fibrillation (*p* > 0.05 for all). These findings suggest that apart from age, the two groups were comparable in their baseline clinical and demographic characteristics.

### 3.2. Neuropsychological and Neuroradiological Features

Neuropsychological assessments demonstrated significantly poorer cognitive performance in participants with mild cognitive impairment (Group II) compared with those without cognitive impairment (Group I). Specifically, Group II exhibited lower scores on both the Mini-Mental State Examination, 2nd Edition (MMSE-2), and the Montreal Cognitive Assessment (MoCA). The median MMSE-2 score in Group II was 23.0 (IQR: 22–24) versus 27.0 (IQR: 26–29) in Group I (*p* < 0.001), while the MoCA score was 21.0 (IQR: 20–24) in Group II compared with 26.0 (IQR: 24–28) in Group I (*p* < 0.001). Depressive symptoms were also more pronounced in Group II, with a median Geriatric Depression Scale (GDS-15) score of 8.8 (IQR: 7.6–9.9) compared with 7.8 (IQR: 6.8–8.7) in Group I (*p* = 0.02). MRI-based neuroradiological assessment using the Fazekas scale revealed a significantly greater burden of white matter hyperintensities (WMHs) in Group II, with a median Fazekas score of 3.0 (IQR: 2.0–3.0) versus 2.0 (IQR: 1.0–3.0) in Group I (*p* = 0.03). Moreover, 55% of participants in Group II were classified as having Grade 3 lesions compared with 27% in Group I (*p* = 0.02).

These findings indicate that mild cognitive impairment is associated with reduced cognitive performance, increased depressive symptomatology, and a higher burden of cerebrovascular pathology.

### 3.3. Transcranial Doppler Hemodynamics

Transcranial Doppler (TCD) ultrasonography revealed significant differences in cerebrovascular flow dynamics between cognitively unimpaired individuals and those with mild cognitive impairment.

Group II demonstrated significantly reduced MBFV compared with Group I (51.6 cm/s [95% CI: 50.8–52.6] vs. 56.3 cm/s [95% CI: 53.6–62.5], *p* < 0.001), suggesting impaired cerebral perfusion in the context of early cognitive decline. A similar pattern was observed for EDV, which was lower in Group II (36.3 cm/s [IQR: 33.4–39.3]) compared with Group I (40.65 cm/s [IQR: 38.07–42.82], *p* = 0.04).

Conversely, indices of vascular resistance were significantly elevated in the cognitively impaired group. The PI was markedly higher in Group II (0.94 [IQR: 0.91–0.96]) compared with Group I (0.73 [IQR: 0.66–0.81], *p* < 0.001). Similarly, the RI was increased in Group II (0.58 [IQR: 0.55–0.65]) relative to Group I (0.51 [IQR: 0.45–0.54], *p* = 0.002), indicating greater cerebrovascular resistance in patients with mild CI.

No statistically significant difference was found in PSV between groups (86.4 cm/s [IQR: 84.9–88.6] in Group II vs. 84.95 cm/s [IQR: 83.82–85.89] in Group I, *p* = 0.15).

Overall, these findings suggest that patients with mild cognitive impairment exhibit a distinct TCD profile characterized by reduced flow velocities and increased resistance, consistent with cerebral small vessel dysfunction.

### 3.4. Predictors of Mild Cognitive Impairment

To identify clinical, neuroimaging, and hemodynamic predictors of mild cognitive impairment, both univariate and multivariate logistic regression analyses were performed.

#### 3.4.1. Univariate Logistic Regression

Univariate analysis revealed that younger age was associated with a lower risk of cognitive impairment (OR = 0.88, 95% CI: 0.79–0.97, *p* = 0.01), while higher scores on the Fazekas visual scale (OR = 2.17, 95% CI: 1.07–1.41, *p* = 0.03) and greater global depression assessment (GDS-15) scores (OR = 1.66, 95% CI: 1.04–2.66, *p* = 0.03) were positively associated with the presence of mild CI.

Among the transcranial Doppler (TCD) measures, several parameters demonstrated significant associations: lower MBFV (OR = 0.64, 95% CI: 0.49–0.84, *p* < 0.01) and lower EDV (OR = 0.78, 95% CI: 0.66–0.91, *p* < 0.01) were protective, while higher RI (OR = 3.4, 95% CI: 2.97–3.88, *p* = 0.02), PI (OR = 0.79, 95% CI: 0.63–1.00, *p* = 0.04), and PSV (OR = 1.32, 95% CI: 1.03–1.69, *p* = 0.02) were associated with increased odds of cognitive impairment.

These findings are detailed in Table 2, which presents the odds ratios, 95% confidence intervals, and *p*-values from the univariate logistic regression.

#### 3.4.2. Multivariate Logistic Regression

**Figure 3:**
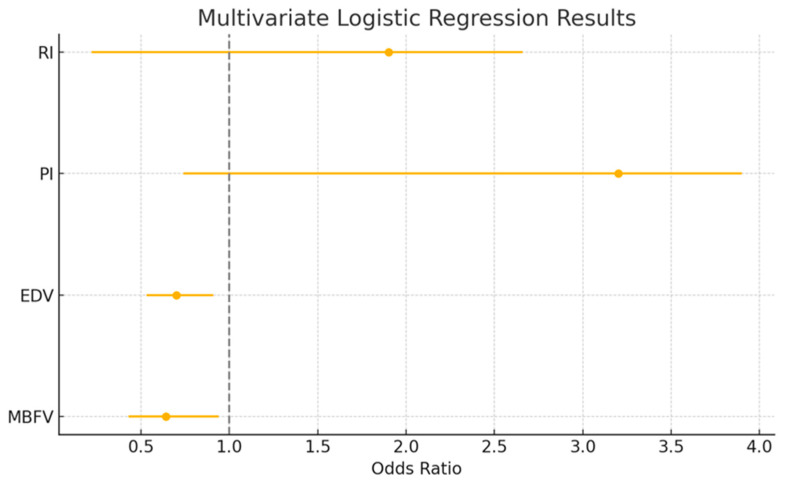
Forest plot showing the odds ratios and 95% confidence intervals from the multivariate logistic regression analysis. Variables with odds ratios below 1 (MBFV, EDV) suggest a protective effect, while those above 1 (PI, RI) indicate increased odds. The vertical line at OR = 1 represents the null effect. Abbreviations: MBFV: mean blood flow velocity; PI: pulsatility index; RI: resistivity index; EDV: end-diastolic blood flow velocity.

To adjust for potential confounders and assess independent effects, variables that reached significance in the univariate model were entered into a multivariate logistic regression analysis. In the multivariate model, MBFV (OR = 0.64, 95% CI: 0.43–0.94, *p* = 0.02), EDV (OR = 0.70, 95% CI: 0.53–0.91, *p* = 0.03), PI (OR = 3.2, 95% CI: 0.74–3.9, *p* < 0.01), and RI (OR = 1.9, 95% CI: 0.22–2.66, *p* < 0.01) emerged as independent predictors of mild CI. These results are also presented in Table 2 and are visually presented in the forest plot (Figure 3), where odds ratios <1 (MBFV, EDV) indicate protective factors, and those >1 (PI, RI) denote increased risk.

### 3.5. Diagnostic Accuracy: ROC Curve Analysis

To further evaluate the discriminative performance of the TCD parameters, ROC curve analyses were conducted.

**Figure 4:**
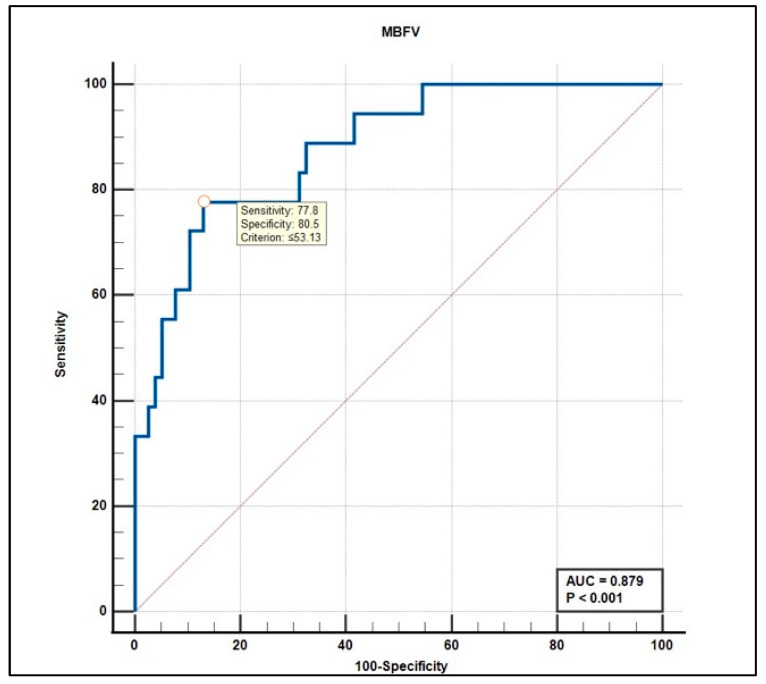
Receiver operating characteristic (ROC) curve for mean blood flow velocity (MBFV) in predicting mild cognitive impairment. The optimal cutoff value of ≤53.13 cm/s yielded a sensitivity of 77.8% and specificity of 80.5%. The area under the curve (AUC) was 0.879 (*p* < 0.001), indicating strong discriminative performance.

MBFV demonstrated the highest accuracy (AUC = 0.879, 95% CI 0.79–0.93, *p* < 0.001), with an optimal cutoff of ≤53.13 cm/s yielding 77.8% sensitivity and 80.5% specificity (Figure 4).

**Figure 5:**
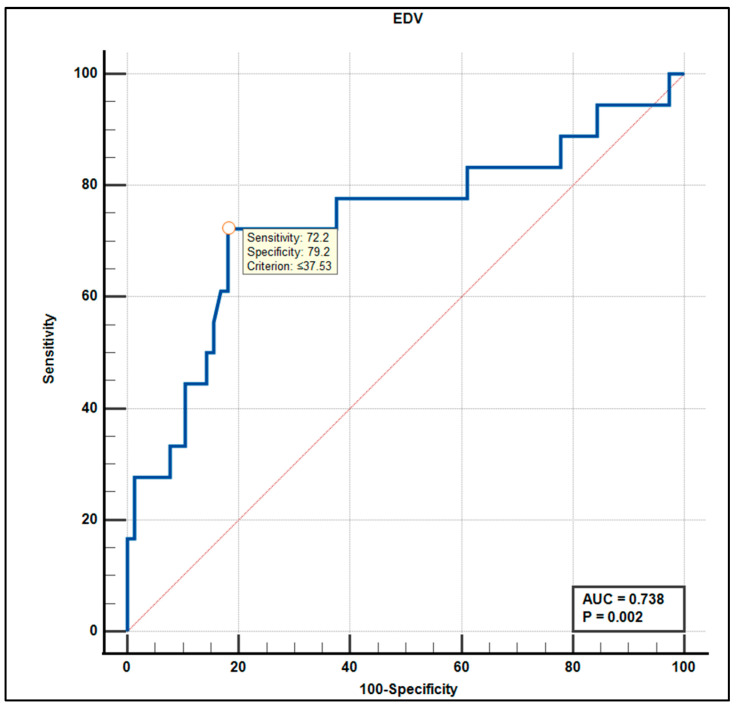
Receiver operating characteristic (ROC) curve for end-diastolic velocity (EDV) in predicting mild cognitive impairment. The optimal cutoff value (≤37.53 cm/s) yielded a sensitivity of 72.2% and specificity of 79.2%. The area under the curve (AUC) was 0.738 (*p* = 0.002), indicating good discriminatory ability.

RI had the strongest overall performance (AUC = 0.919, 95% CI: 0.84–0.96, *p* < 0.001), achieving near-perfect separation at a specific threshold (Figure 5).

**Figure 6:**
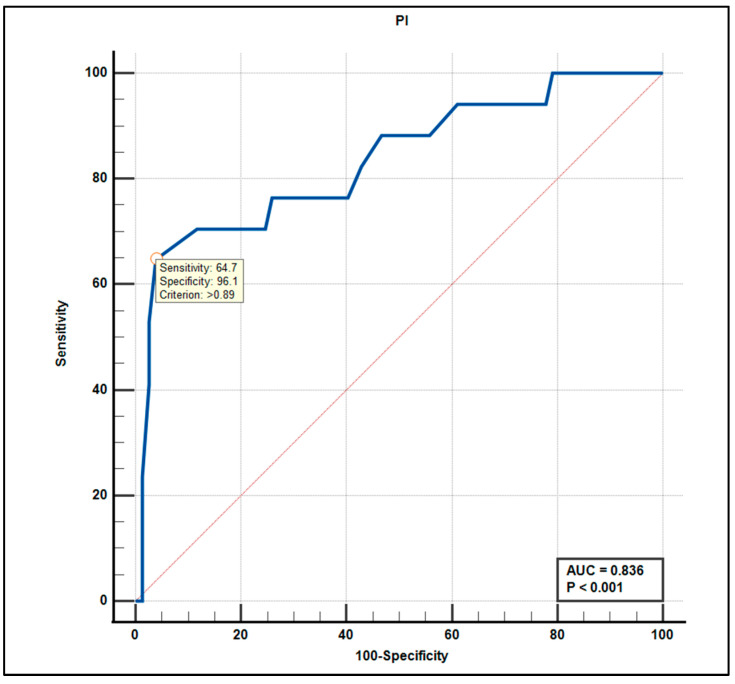
Receiver operating characteristic (ROC) curve for the pulsatility index (PI) in the prediction of mild cognitive impairment. The optimal cutoff value of >0.89 yielded a sensitivity of 64.7% and specificity of 96.1%. The area under the curve (AUC) was 0.836 (*p* < 0.001), indicating high diagnostic accuracy.

PI also performed well (AUC = 0.836, 95% CI: 0.74–0.90, *p* < 0.001), with a cutoff of >0.89 yielding 64.7% sensitivity and 96.1% specificity (Figure 6).

**Figure 7:**
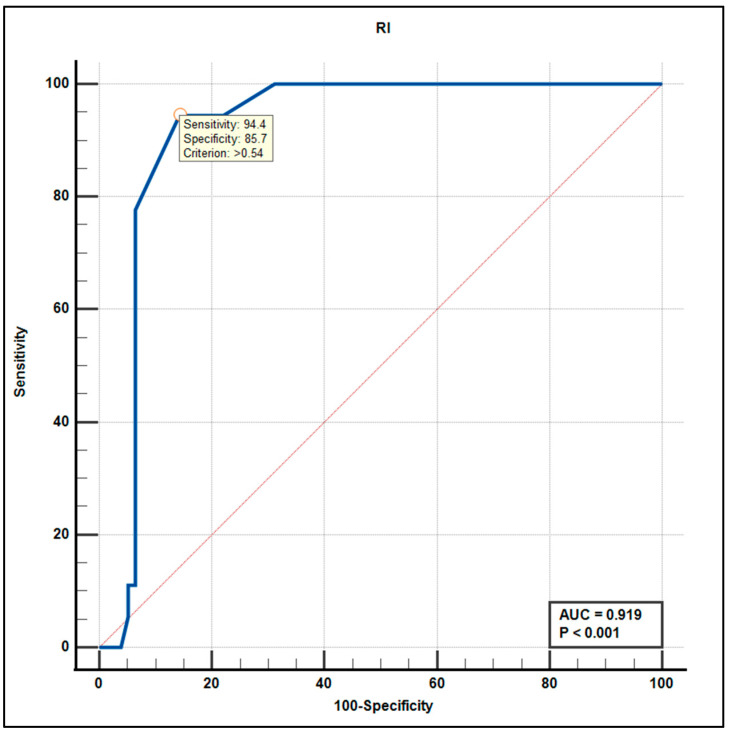
Receiver operating characteristic (ROC) curve of the resistive index (RI) for predicting mild cognitive impairment. The model achieved an area under the curve (AUC) of 0.919 (*p* < 0.001), indicating excellent diagnostic performance. The optimal cutoff point provided high sensitivity and specificity (values indicated on the curve).

EDV showed moderate accuracy (AUC = 0.738, 95% CI:0.63–0.82, *p* = 0.002), with optimal sensitivity and specificity at ≤37.53 cm/s (Figure 7).

**Figure 8:**
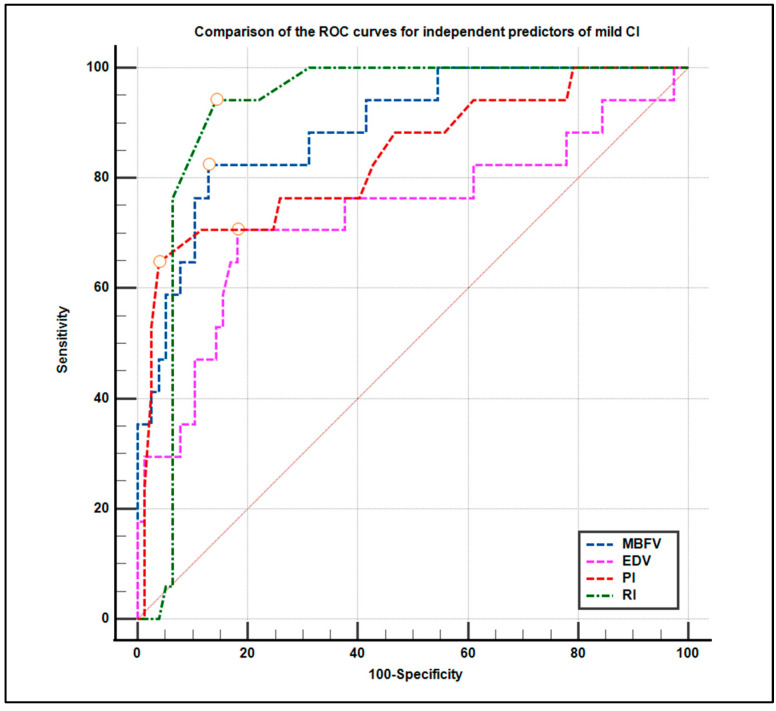
Comparison of the receiver operating characteristic (ROC) curves for independent predictors of mild cognitive impairment. The figure illustrates the diagnostic performance of the mean blood flow velocity (MBFV), end-diastolic velocity (EDV), pulsatility index (PI), and resistive index (RI). Each curve reflects the sensitivity and specificity trade-offs at various thresholds. MBFV and RI demonstrated the highest discriminative power, with area under the curve (AUC) values indicating excellent diagnostic accuracy.

A comparative ROC curve of all four predictors (Figure 8) confirmed that MBFV and RI had the highest diagnostic performance, followed by PI and EDV.

## 4. Discussion

The present study demonstrates that altered cerebral hemodynamics—specifically reduced mean blood flow velocity (MBFV), lower end-diastolic velocity (EDV), and elevated resistive and pulsatility indices (RI, PI)—are strongly associated with mild cognitive impairment (MCI) and greater depressive symptom severity in older adults. These findings, drawn from a multimodal approach combining neuropsychological testing, MRI-based white matter lesion (WML) grading, and transcranial Doppler (TCD) ultrasonography, provide new evidence for the utility of non-invasive cerebrovascular markers in early cognitive and affective assessment.

Our analysis revealed that RI and MBFV were the most accurate TCD-derived predictors of MCI, with RI achieving an AUC of 0.919 and MBFV an AUC of 0.879. These parameters not only differentiated cognitively impaired participants from the controls, but also correlated with a greater WML burden and depressive symptoms. Importantly, our results identified RI as a novel, independent predictor of MCI, suggesting that increased cerebrovascular resistance may reflect underlying small vessel pathology linked to neurodegeneration.

In contrast, EDV showed moderate diagnostic accuracy (AUC = 0.738), while PI offered high specificity (96.1%) at the cost of lower sensitivity. The strength of these hemodynamic markers lies in their potential clinical applicability: TCD is portable, cost-effective, and accessible—making it a valuable tool in primary care or resource-limited settings.

Crucially, we found that participants with MCI exhibited a higher prevalence of Grade 3 WMLs on MRI, reinforcing the relationship between small vessel disease and cognitive decline. Moreover, the association between elevated RI and PI with higher Faze-kas scores strengthens the case for a structural-functional link that TCD may help to monitor in vivo. Interestingly, no significant group differences were observed in conventional vascular risk factors (e.g., hypertension, diabetes), highlighting that hemodynamic impairments may precede or act independently of traditional cardiovascular risk profiles.

By incorporating depressive symptoms into our analysis, we further support a vascular-affective-cognitive framework in late-life mental health. GDS-15 scores correlated with key TCD parameters, suggesting that neurovascular dysfunction may underlie both cognitive and mood disturbances. This multidomain approach aligns with emerging models of vascular depression and cognitive impairment as intertwined outcomes of cerebrovascular compromise.

These findings hold important clinical implications: the early identification of at-risk individuals through bedside vascular imaging could enhance prevention strategies and inform personalized interventions targeting cerebrovascular health.

While numerous studies have individually explored the association between cognitive decline and white matter hyperintensities [6,27], or separately investigated TCD parameters as early vascular biomarkers [3,4,5], few have examined the interplay between these modalities in a clinically stratified population. The practical value of TCD lies in its non-invasive, low-cost, and bedside applicability—making it particularly useful in outpatient geriatric or primary care settings where access to MRI may be limited.

This multidimensional design adds originality to the current work and strengthens its clinical relevance in early-stage vascular cognitive impairment. In comparison with previous studies, our findings corroborate and extend the understanding of the role of cerebrovascular dysfunction in MCI. Consistent with Sabayan et al. [28,29] and Vicenzini et al. [12], we observed reduced mean blood flow velocity and an elevated pulsatility index in patients with cognitive impairment, suggesting impaired microvascular compliance and autoregulation. We also demonstrated that lower MBFV and EDV were associated with poorer MoCA and GDS-15 scores, reinforcing the link between reduced cerebral perfusion and neuropsychiatric burden. These results are in line with prior work by Sabayan et al. [28,29] and Taylor et al. [30], which associated reduced cerebral blood flow with cognitive decline and depression. Lim et al. [31] further support the clinical value of TCD markers for predicting conversion from MCI to Alzheimer’s disease.

The present study evaluated the diagnostic utility of various TCD parameters using ROC curve analysis. Among the parameters assessed, mean blood flow velocity (MBFV) and resistance index (RI) emerged as the most robust discriminators.

MBFV demonstrated a high diagnostic accuracy (AUC = 0.879, *p* < 0.001), with an optimal cut-off value of ≤53.13 cm/s, yielding a 77.8% sensitivity and 80.5% specificity. This threshold effectively differentiates between affected and non-affected individuals, offering a practical marker for clinical evaluation.

In contrast, end-diastolic velocity (EDV) displayed moderate accuracy (AUC = 0.738, *p* = 0.002), with an optimal cut-off of ≤37.53 cm/s balancing sensitivity and specificity. Although its discriminative power was lower compared with other parameters, EDV may still offer supportive diagnostic information when used in combination.

The RI showed the strongest overall performance (AUC = 0.919, *p* < 0.001), with an optimal cut-off value of >0.54%, yielding a 94.4% sensitivity and 85.7% specificity. The steep slope of the ROC curve suggests a well-defined threshold capable of high diagnostic precision. Our research identified RI as an independent predictor of MCI, which has been inconsistently reported in earlier research. Krejza et al. [32] reported mixed associations between RI and cognitive performance, suggesting the need for context-specific interpretation. By demonstrating the superior predictive accuracy of the RI over other TCD parameters, our findings offer new insights into the utility of this metric as a non-invasive, bedside tool for early cognitive screening. Our results suggest that RI may serve as a sensitive marker of increased vascular resistance linked to neurodegeneration.

The PI also contributed significantly to classification accuracy (AUC = 0.836, *p* < 0.001). A cut-off value of >0.89 provided a 64.7% sensitivity and an excellent 96.1% specificity, indicating its strength as a confirmatory tool where high specificity is required.

The comparative ROC analysis reaffirmed the superior performance of MBFV and RI, followed by PI and EDV. These findings underscore the importance of parameter-specific thresholds in optimizing the clinical application of TCD in diagnostic workflows. The identification of MBFV and RI as reliable and independent predictors of MCI offers practical diagnostic value, especially in settings where advanced imaging (e.g., MRI) may be limited. Furthermore, the ability to combine TCD findings with neuropsychological assessments enhances diagnostic precision and risk stratification, particularly in populations at risk for vascular cognitive impairment.

The high prevalence of Grade 3 white matter lesions (WMLs) in the MCI group supports earlier literature suggesting a strong association between small vessel disease and cognitive decline [8,33,34,35]. However, the integration of TCD data in this context is less frequently reported. By showing that elevated PI and RI co-occur with higher Fazekas scores, we reinforce the concept that these indices may reflect the underlying structural pathology. This aligns with findings by Kidwell et al. [36] but adds depth by including hemodynamic correlates.

Additionally, our data showed no significant differences in conventional vascular risk factors (e.g., hypertension, diabetes) between groups, consistent with some studies [37] but differing from others that found hypertension to be a key driver of cerebral small vessel disease [38]. This discrepancy may be due to the relatively small sample size or the presence of subclinical vascular changes not captured by binary risk factor categorization, emphasizing the importance of functional imaging techniques like TCD.

The integration of depressive symptoms (as measured by GDS-15) into this vascular-cognitive model also supports a multi-domain assessment approach. Given the bidirectional relationships between depression and cognitive impairment in late life, incorporating affective symptomatology into cerebrovascular evaluations may improve early detection and guide more holistic interventions.

Our findings add to the growing body of evidence that cerebrovascular health plays a crucial role in the maintenance of cognitive function and emotional well-being in older adults. These results also support the conceptualization of vascular cognitive impairment and vascular depression as clinical entities arising from shared vascular pathology [39,40,41]. This supports a shift toward multimodal assessment strategies for identifying high-risk individuals and tailoring early interventions. The clinical implications of our findings are significant. TCD is a non-invasive, cost-effective, and accessible bedside tool that could be implemented in routine geriatric and neurology clinics to help detect early vascular contributions to cognitive impairment. Future longitudinal studies are needed to validate whether the identified TCD markers—particularly RI and MBFV—predict progression from MCI to dementia and confirm their role in disease trajectory. Given the single-country, single-ethnicity sample, our findings may not fully generalize to other populations. Multicenter studies across diverse ethnic and geographic cohorts are needed.

### Study Limitations

Despite its strengths, this study had several limitations. First, its cross-sectional de-sign restricted the ability to infer causality. While significant associations were observed between hemodynamic parameters and cognitive status, future longitudinal studies are essential to evaluate whether these TCD indices can reliably predict cognitive decline or progression to dementia over time. Second, the sample size—particularly the mild cognitive impairment (MCI) subgroup—was relatively small (*n* = 18), which may reduce the statistical power and limit generalizability. Expanding the cohort in future research, especially to include a larger number of MCI cases, will be critical for validating these findings across diverse populations. Third, although the Fazekas scale provides valuable insight into white matter lesion burden, it is a semi-quantitative measure. Incorporating advanced neuroimaging techniques such as volumetric MRI or diffusion tensor imaging (DTI) could offer more precise assessments of white matter integrity. Additionally, genetic or inflammatory biomarkers such as APOE genotype or CRP levels were not assessed in the current study, limiting mechanistic interpretation. While associations between hemodynamic indices and cognitive status were observed, longitudinal data are needed to determine whether these TCD markers predict cognitive decline or progression to dementia.

## 5. Conclusions

This study highlights significant associations between altered cerebral hemodynamics and both cognitive and affective symptoms in older adults. Lower blood flow velocities and alterations in vascular resistance, as measured by transcranial Doppler ultrasonography, were reliable, non-invasive markers for the early detection of MCI in older adults. By providing a detailed hemodynamic profile correlated with neuropsychological outcomes and structural MRI markers, this study offers practical insight into the use of TCD as a non-invasive, bedside tool in the clinical assessment of vascular brain aging. Future longitudinal and interventional studies are warranted to validate these findings and assess whether targeting cerebrovascular health can mitigate cognitive and emotional decline in older populations. Our future research will incorporate longitudinal follow-up to establish the predictive validity of TCD parameters, integrate multimodal biomarkers such as diffusion tensor imaging (DTI) and perfusion MRI, and examine genetic (e.g., APOE status) and inflammatory profiles to validate these findings and assess whether targeting cerebrovascular health can mitigate cognitive and emotional decline in older adults.

## Figures and Tables

**Table 1:** Clinical-demographic characteristics, neuroradiological features, neuropsychological test scores, and TCD values of all participants.

Parameter	Group I (No Cognitive Impairment) *n* = 78	Group II (Mild Cognitive Impairment) *n* = 18	All Patients *n* = 96	*p* Value
**Demographic and clinical characteristics**				
Age, mean ± SD (years)	68.8 ± 5.8	72.5 ± 5.2	71.8 ± 5.5	0.01
Male sex *n* (%)	39 (50)	10 (55)	49 (51)	0.70
Educational level, mean ± SD (years)	8.0 ± 3.4	6.8 ± 3.9	8.0 ±3.5	0.24
Smokers *n* (%)	18 (23)	5 (25)	23 (23.9)	0.85
Hypertension *n* (%)	63 (81)	15 (83)	78 (81.2)	0.84
Hypercholesterolemia *n* (%)	27 (34)	6 (35)	33 (34)	0.93
Diabetes *n* (%)	17 (21)	4 (22)	21 (21.8)	0.92
Coronaropathy *n* (%)	11 (14)	3 (15)	14 (14.5)	0.91
Atrial fibrillation *n* (%)	11 (14)	3 (15)	14 (14.5)	0.91
**Neuropsychological and imaging data**				
MMSE-2, median (IQR)	27.0 (26.0–29.0)	23.0 (22–24)	27.0 (25.2–29.0)	<0.001
MoCA (I–III quartile)	26.0 (24.0–28.0)	21.0 (20–24)	23.0 (21.0–25.0)	<0.001
GDS-15 median (I–III quartile)	7.8 (6.8–8.7)	8.8 (7.6–9.9)	7.9 (6.9–8.8)	0.02
MRI Fazekas visual scale	2.0 (1.0–3.0)	3.0 (2.0–3.0)	2.0 (1.0–3.0)	0.03
Grade 1 *n* (%)	27 (35)	3 (17	30 (31)	0.14
Grade 2 *n* (%)	30 (38)	5 (28)	35 (37	0.42
Grade 3 *n* (%)	21 (27)	10 (55)	31 (32)	0.02
**TCD parameters**				
Mean BFV	56.3 (53.6–62.5)	51.6 (50.8–52.6)	56.3 (53.6–62.5)	<0.001
PI (%) median (I–III quartile)	0.73 (0.66–0.81)	0.94 (0.91–0.96)	0.73 (0.66–0.81)	<0.001
RI (%) median (I–III quartile)	0.51 (0.45–0.54)	0.58 (0.55–0.65)	0.51 (0.45–0.54)	0.002
PSV (cm/s) median (I–III quartile)	84.95 (83.82–85.89)	86.4 (84.9–88.6)	84.9 (84.02–86.32)	0.15
EDV (cm/s) median (I–III quartile)	40.65 (38.07–42.82)	36.3 (33.4–39.3)	39.9 (36.91–42.67)	0.04

Abbreviations: SD, standard deviation; GDS-15, 15-item Geriatric Depression Scale; MMSE-2, Mini-Mental State Examination, 2nd Edition; MoCA, Montreal Cognitive Assessment; MRI, magnetic resonance imaging; PI, pulsatility index; RI, resistivity index; PSV, peak systolic blood flow velocity; EDV, end-diastolic blood flow velocity; TCD, transcranial Doppler hemodynamics.

**Table 2:** Predictors for mild cognitive impairment.

Univariate Logistic Regression	Odds Ratio	95% CI	*p* Value
Age (years)	0.88	0.79–0.97	0.01
GDS-15	1.66	1.04–2.66	0.03
MRI Fazekas visual scale	2.17	1.07–1.41	0.03
MBFV (cm/s)	0.64	0.49–0.84	<0.01
PSV (cm/s)	1.32	1.03–1.69	0.02
EDV (cm/s)	0.78	0.66–0.91	<0.01
PI (%)	0.79	0.63–1.00	0.04
RI (%)	3.4	2.97–3.88	0.02
**Multivariate Logistic Regression**	**Odds Ratio**	**95% CI**	***p* Value**
MBFV (cm/s)	0.64	0.43–0.94	0.02
EDV (cm/s)	0.70	0.53–0.91	0.03
PI (%)	3.2	0.74–3.9	<0.01
RI (%)	1.9	0.22–2.66	<0.01

Abbreviations: MRI, magnetic resonance imaging; GDS-15, 15-item Geriatric Depression Scale; MBFV, mean blood flow velocity; PI, pulsatility index; RI, resistivity index; PSV, peak systolic blood flow velocity; EDV, end-diastolic blood flow velocity.

## Data Availability

The data are available upon request to the first author, S.-F.A.
